# The Rice *SPOTTED LEAF4* (*SPL4*) Encodes a Plant Spastin That Inhibits ROS Accumulation in Leaf Development and Functions in Leaf Senescence

**DOI:** 10.3389/fpls.2018.01925

**Published:** 2019-01-07

**Authors:** Giha Song, Choon-Tak Kwon, Suk-Hwan Kim, Yejin Shim, Chaemyeong Lim, Hee-Jong Koh, Gynheung An, Kiyoon Kang, Nam-Chon Paek

**Affiliations:** ^1^Department of Plant Science, Plant Genomics and Breeding Institute, Research Institute of Agriculture and Life Sciences, Seoul National University, Seoul, South Korea; ^2^Department of Plant Molecular Systems Biotechnology, Crop Biotech Institute, Kyung Hee University, Seoul, South Korea

**Keywords:** spastin, microtubule severing protein, lesion mimic mutant, rice (*Oryza sativa*), senescence, reactive oxygen species

## Abstract

Lesion mimic mutants (LMMs) are usually controlled by single recessive mutations that cause the formation of necrotic lesions without pathogen invasion. These genetic defects are useful to reveal the regulatory mechanisms of defense-related programmed cell death in plants. Molecular evidence has been suggested that some of LMMs are closely associated with the regulation of leaf senescence in rice (*Oryza sativa*). Here, we characterized the mutation underlying *spotted leaf4* (*spl4*), which results in lesion formation and also affects leaf senescence in rice. Map-based cloning revealed that the γ ray-induced *spl4-1* mutant has a single base substitution in the splicing site of the *SPL4* locus, resulting in a 13-bp deletion within the encoded microtubule-interacting-and-transport (MIT) spastin protein containing an AAA-type ATPase domain. The T-DNA insertion *spl4-2* mutant exhibited spontaneous lesions similar to those of the *spl4-1* mutant, confirming that *SPL4* is responsible for the LMM phenotype. In addition, both *spl4* mutants exhibited delayed leaf yellowing during dark-induced or natural senescence. Western blot analysis of *spl4* mutant leaves suggested possible roles for SPL4 in the degradation of photosynthetic proteins. Punctate signals of SPL4-fused fluorescent proteins were detected in the cytoplasm, similar to the cellular localization of animal spastin. Based on these findings, we propose that SPL4 is a plant spastin that is involved in multiple aspects of leaf development, including senescence.

## Introduction

Among the defense mechanisms activated in response to pathogen attacks in plants, the hypersensitive response (HR), which induces rapid death of infected cells, prevents the spread of pathogens to adjacent cells (Morel and Dangl, 1997; Takahashi et al., 2009). Lesion mimic mutants (LMMs) exhibit spontaneous cell death in the absence of pathogen attacks and have been isolated from plant species including barley (*Hordeum vulgare*) (Wolter et al., 1993), maize (*Zea mays*) (Hoisington et al., 1982), tomato (*Solanum lycopersicum*) (Badel et al., 2006), Arabidopsis (*Arabidopsis* *thaliana*) (Dietrich et al., 1994), and rice (*Oryza sativa*) (Kim et al., 2009). The autonomous lesions in LMMs tend to be accompanied by excessive levels of reactive oxygen species (ROS), which lead to accelerated cell death (Van Breusegem and Dat, 2006).

Based on the lesion phenotype of LMMs, some of the underlying genes have been cloned and functionally characterized. A spotted leaf gene *Spl7* encodes a heat shock protein and its mutation is responsible to lesion formation in the rice leaves (Yamanouchi et al., 2002). Mutation of *SPL5* encoding a putative splicing factor 3b subunit 3 (SF3b3) continuously developed small reddish-brown necrotic lesions on the rice leaves (Chen et al., 2012). The probenazole-induced protein (PBZ1) of which expression is ectopically induced in *spl1* mutant is localized in theseed aleurone layer and associated with programmed cell death (PCD) (Kim et al., 2008). Impairment of COPROPORPHYRINOGEN III OXIDASE (CPOX) in the *rice lesion initiation 1* (*rlin1*) mutant causes the formation of necrotic lesions in rice leaves and stems due to excessive ROS accumulation (Sun et al., 2011). *SPL11* encodes a U-box/armadillo repeat protein conferring E3 ubiquitin ligase activity and the *spl11* mutant displays a spontaneous cell death phenotype and enhanced resistance to fungal and bacterial diseases in rice (Zeng et al., 2004).

Recent studies have reported that a few of LMMs are associated with the regulatory pathways of leaf senescence. *SPOTTED LEAF3* (*SPL3*) encodes MITOGEN-ACTIVATED PROTEIN KINASE KINASE KINASE1 (MAPKKK1), and the *spl3* mutant causes to cell death due to lack of ROS scavenging activity (Wang et al., 2015). This mutant also exhibits delayed abscisic acid-mediated leaf senescence. On the contrary, the mutation of *SPL28*, encoding CLATHRIN-ASSOCIATED ADAPTOR PROTEIN COMPLEX 1 MEDIUM SUBUNIT μ1 (AP1M1), promotes leaf yellowing during senescence (Qiao et al., 2010). Most recently, Wang et al. (2017) have found that *SPL33* encodes a eukaryotic translation elongation factor 1 alpha (eEF1A)-like protein. The *spl33* mutant exhibits both phenotype of PCD-mediated cell death and early senescence. Mutation of *spotted leaf sheath* (*sles*) encoding a putative expressed protein containing kinase domain exhibits precocious senescence (Lee et al., 2018).

Notably, among more than 40 LMMs which have been reported in rice (Wu et al., 2008), mutation of the *LESION MIMIC RESEMBLING* (*LMR*) locus encoding a microtubule interacting and transport (MIT) protein causes the LMM phenotype along with excess ROS accumulation (Fekih et al., 2015). Microtubules (MTs) are dynamic cytoskeletal polymers that play essential roles in cell division (de Keijzer et al., 2014), morphogenesis (Mathur and Hülskamp, 2002), and cell migration (Villari et al., 2015). In the plant cell, MTs coordinate the deposition of cellulose microfibrils in the cell wall, which affects growth and development in plants. They also play key roles in the responses to hormones, pathogens, and environmental stresses (Buschmann and Lloyd, 2008).

MT arrays are reorganized according to the need of cells in response to internal cues and external stimuli (Shibaoka, 1994). This plasticity includes MT growth, stabilization, destabilization, and interaction with other cellular organelles (Wade, 2009), and is regulated by interactions with MT-associated proteins (MAPs), and MT-severing proteins (Gouveia and Akhmanova, 2010; Díaz-Valencia et al., 2011). Three classes of MT-severing proteins have been identified; katanin (McNally and Vale, 1993), spastin (Hazan et al., 1999), and fidgetin (Cox et al., 2000). They all have a highly conserved C-terminal AAA domain that contributes to the formation of hexameric rings or dodecameric stacked rings to interact with MTs (Neuwald et al., 1999; Vale, 2000; Lupas and Martin, 2002).

In humans, hereditary spastic paraplegia (HSP) is a devastating neurodegenerative disorder characterized by a progressive spasticity and lower limb weakness (Crosby and Proukakis, 2002). However, approximately 40% of HSP diseases are caused by the malfunction of the spastin protein, SPASTIC PARAPLEGIA 4 (SPG4) (Hazan et al., 1999; Fonknechten et al., 2000). Unlike the many studies of animal spastin implicated in this severe hereditary disease, little is known about the functions of plant spastins, even though putative spastin proteins are conserved in plants (Fekih et al., 2015).

Here, we characterized the rice *spl4-1* LMM, which shows autonomous lesions accompanied by ROS accumulation in the leaf blades. By map-based cloning, we found that the *SPL4* locus encodes a MT-interacting-and-transport spastin protein containing an AAA-type ATPase domain. Mutation of *SPL4* resulted in delayed senescence: photosynthetic proteins remained abundant in the detached leaves under dark-induced senescence conditions. Confocal microscopic observation implied that SPL4 is localized to the cytoplasm. Collectively, our results point to a novel function for plant spastin in the inhibition of lesion formation in the leaves during vegetative growth and promotion of leaf yellowing during senescence.

## Materials and Methods

### Plant Materials, Growth Conditions, and Dark Treatment

The *spl4-1* mutant was previously isolated from a mutant pool produced by γ-ray irradiation to the Japanese *japonica* rice (*Oryza sativa*) cultivar “Norin 8” (Iwata et al., 1978). The T-DNA insertional knockout mutant of *SPL4* (LOC_Os06g03940; PFG_3A-16679, designated as *spl4-2*) was derived from the Korean *japonica* rice cultivar “Dongjin”^1^ (Jeon et al., 2000; Jeong et al., 2006). Rice plants were cultivated in the paddy field under natural long day (NLD) conditions ( ≥ 14 h light/day, 37°N latitude, Suwon, Korea). For the dark treatment, the detached leaves of rice plants grown in the paddy field under NLD conditions were incubated on 3 mM MES (pH 5.8) buffer with the abaxial side up at 28°C in complete darkness. The leaf disks were sampled at the specified DDI for each experiment.

### Detection of Reactive Oxygen Species (ROS)

Hydrogen peroxide (H_2_O_2_) in the rice leaves was detected using 3,3’-diaminobenzidine (DAB) as previously described (Kim et al., 2018). The rice leaves grown in the paddy field under NLD conditions were sampled at 60, 70, 80, and 125 days after sowing (DAS) and incubated in 0.1% (w/v) DAB (Sigma) solution for 12 h at 28°C with gentle shaking (40 rpm). Chlorophyll was then completely removed by incubation in 90% ethanol at 80°C. H_2_O_2_ was visualized as reddish-brown stains. Detection of singlet oxygen (^1^O_2_) was conducted as previously described with some modifications (Kwon et al., 2017). The rice leaves grown in the paddy field for 133 DAS were vacuum infiltrated with 10 mM sodium phosphate buffer (pH 7.5) containing 50 μM Singlet Oxygen Sensor Green reagent (SOSG, Invitrogen). After incubation for 30 min in the dark, the fluorescence emission of SOSG was detected by a laser scanning confocal microscope (Carl Zeiss LSM510). The excitation and emission wavelengths were 480 and 520 nm, respectively. The red autofluorescence emission from chlorophyll was detected following excitation at 543 nm.

### Map-Based Cloning

The *SPL4* locus was previously mapped to the short arm of chromosome 6 (Iwata et al., 1978). In this study, a mapping population of 798 F_2_ individuals was generated by crossing the *japonica*-type *spl4-1* mutant and the tongil-type Milyang23 (an *indica*/*japonica* hybrid cultivar, M23). To determine the chromosomal localization of the *SPL4* locus, we initially performed a small-scale mapping using 100 *spl4* homozygous F_2_ plants, 11 simple sequence repeat (SSR) markers, and 5 sequence-tagged site (STS) markers distributed on chromosome 6 (Supplementary Table S1). The SSR marker information is available in GRAMENE^2^. For fine mapping, 8 additional STS markers were designed by comparing the genomic DNA sequences of the *spl4-1* mutant with those of the M23 cultivar (Supplementary Table S1). Using these 8 STS markers and the 198 F_3_ individuals with the spontaneous lesion phenotype, the *SPL4* locus was fine-mapped to the 77-kb region between the STS8, and STS9 markers on chromosome 6.

### Chlorophyll Quantification

To measure the total chlorophyll levels, pigments were extracted from equal fresh weights of leaves with 80% ice-cold acetone. The concentration of total chlorophyll was determined using a UV/VIS spectrophotometer (BioTek) and calculated as previously described (Lichtenthaler, 1987).

### Sodium Dodecyl Sulfate Polyacrylamide Gel Electrophoresis (SDS-PAGE) and Immunoblot Analysis

Total proteins were extracted from the detached leaves of 2-month-old plants that were incubated in complete darkness. Leaf tissue (10 mg) was homogenized with 100 μl of SDS sample buffer [50 mM Tris, pH 6.8, 2 mM EDTA, 10% (w/v) glycerol, 2% SDS, and 6% 2-mercaptoethanol]. Then, 4 μl of each protein extract was subjected to 12% SDS (w/v) PAGE, and the resolved proteins were electroblotted onto an Immobilon-P Transfer Membrane (Millipore). Antibodies against photosynthetic proteins (Lhca2, Lhca3, Lhcb2, Lhcb6, PsaA, and PsbD) and the large subunit of rubisco (RbcL) (Agrisera) were used for immunoblot analysis. Horseradish peroxidase activity of secondary antibodies (Sigma) was detected using the ECL system (WESTSAVE, AbFRONTIER) according to the manufacturer’s instructions.

### Reverse Transcription and Quantitative PCR (RT-qPCR) Analysis

Total RNA was extracted from leaves using a Total RNA Extraction Kit(MGmed) according to the manufacturer’s protocols. First-strand cDNA was synthesized from 2 μg of total RNA in a 100 μl volume using oligo(dT)_15_ primers and M-MLV reverse transcriptase (Promega). The transcript levels of *SPL4* were detected by qPCR using *SPL4*-specific primers (Supplementary Table S1). Rice *UBIQUITIN 5* (*OsUBQ5*) (AK061988) was used as an internal control for normalization (Supplementary Table S1; Jain et al., 2006). The 20– μl total reaction volume included 2 μl of cDNA mixture, 2 μl of 0.5 μM primer, and 10 μl of 2X GoTaq qPCR Master Mix (Promega). PCR was performed with a LightCycler 480 (Roche) using the following conditions: 95°C for 2 min followed by 45 cycles of 95°C for 10 s and 60°C for 1 min.

### Plasmid Construction

The full-length cDNA of *SPL4* was amplified by PCR using gene-specific primers (Supplementary Table S1), and inserted into the pCR8/GW/TOPO vector (Invitrogen). Then, the *SPL4* cDNA was transferred into the pMDC43 and pEarleyGate 104 (pEG104) gateway binary vectors using Gateway LR Clonase II Enzyme Mix (Invitrogen), resulting in *35S::GFP-SPL4*, and *35S::YFP-SPL4* constructs, respectively.

### Subcellular Localization of SPL4

Rice protoplast isolation was carried out as previously described with some modifications (Yoo et al., 2007). The leaf sheaths of 10-day-old etiolated seedlings of the *japonica*-type rice cultivar “Dongjin” were chopped and transferred into a digestion solution [0.5 M mannitol, 10 mM MES (pH 5.7), 1.5% (w/v) Cellulase ONOZUKA R-10 (Yakult, Japan), 0.75% (w/v) Macerozyme R-10 (Yakult, Japan), 0.1% (w/v) BSA, 10 mM CaCl_2_, and 5 mM 2-mercaptoethanol]. After vacuum infiltration for 10 min, the tissues were digested for 4.5 h with gentle shaking (40 rpm) at 28°C. Following the enzymatic digestion, the protoplasts were released with W5 solution [154 mM NaCl, 125 mM CaCl_2_, 5 mM KCl, and 2 mM MES (pH 5.8)]. Then, the protoplasts were adjusted to 10^7^ to 10^8^ cells per 1 ml of MMG solution [0.5 M mannitol, 15 mM MgCl_2_, and 4 mM MES (pH 5.7)] using a hemocytometer. The 50 μl of protoplasts were incubated with 110 μl of PEG solution [0.2 M mannitol, 100 mM CaCl_2_, and 40% (w/v) PEG 4000 (Fluka)] containing 15 μg of plasmids (*35S::GFP-SPL4* or *35S::GFP*) for 15 min in the dark at 28°C. Then, the protoplasts were washed twice with W5 solution and resuspended in 1.5 ml of an incubation solution [0.5 M mannitol, 20 mM KCl, and 4 mM MES (pH 5.7)]. After incubation for 12 h in the dark, the protoplasts were examined by a confocal laser scanning microscope (Carl Zeiss LSM710).

To examine the subcellular localization of SPL4 in onion (*Allium cepa*) epidermal cells, the plasmids (*35S::YFP-SPL4* or *35S::YFP*) were transiently expressed in the onion epidermal cell layers using a DNA Particle Delivery System (Biolistic PDS-1000/He, Bio-Rad). After incubation on a phytoagar plate containing Murashige and Skoog medium for 18 h in the dark at 28°C, the onion cells were observed with a confocal laser scanning microscope (Carl Zeiss LSM710).

## Results

### Characterization of the *spl4-1* Mutant in Rice

**FIGURE 1:**
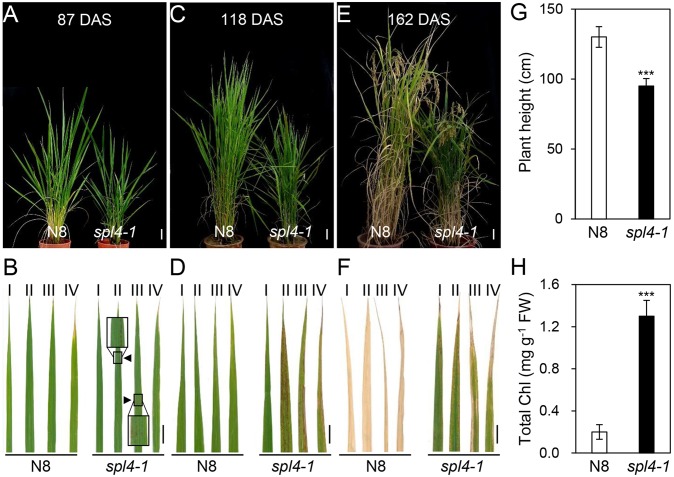
Phenotypic characterization of the *spl4-1* mutant. The pictures of N8 and *spl4-1* plants and leaves were taken from plants grown in the paddy field at 87 days after sowing (DAS) **(A,B)**, 118 DAS **(C,D)**, and 162 DAS **(E,F)**. **(A,C,E)** Plant phenotype of wild-type Norin 8 (N8, *left*) and the *spl4-1* mutant (*right*). White scale bars = 6 cm. **(B,D,F)** Representative leaves of N8 and *spl4-1* plants. The Roman numerals I, II, III, and IV represent the first, second, third, and fourth leaves from the top of the plants, respectively. Black triangles and boxes in **(B)** indicate the location and enlargement of autonomous lesions. Black scale bars = 2 cm. **(G)** The plant height was measured at 162 DAS. **(H)** Total chlorophyll concentration was determined from the first leaves (I) of N8 and *spl4-1* plants shown in **(F)**. Mean and standard deviations were obtained from more than three biological replicates. Asterisks indicate a statistically significant difference between N8 and *spl4-1* plants according to Student’s *t*-test (^∗∗∗^*P* < 0.001). These experiments were repeated twice with similar results.

**FIGURE 2:**
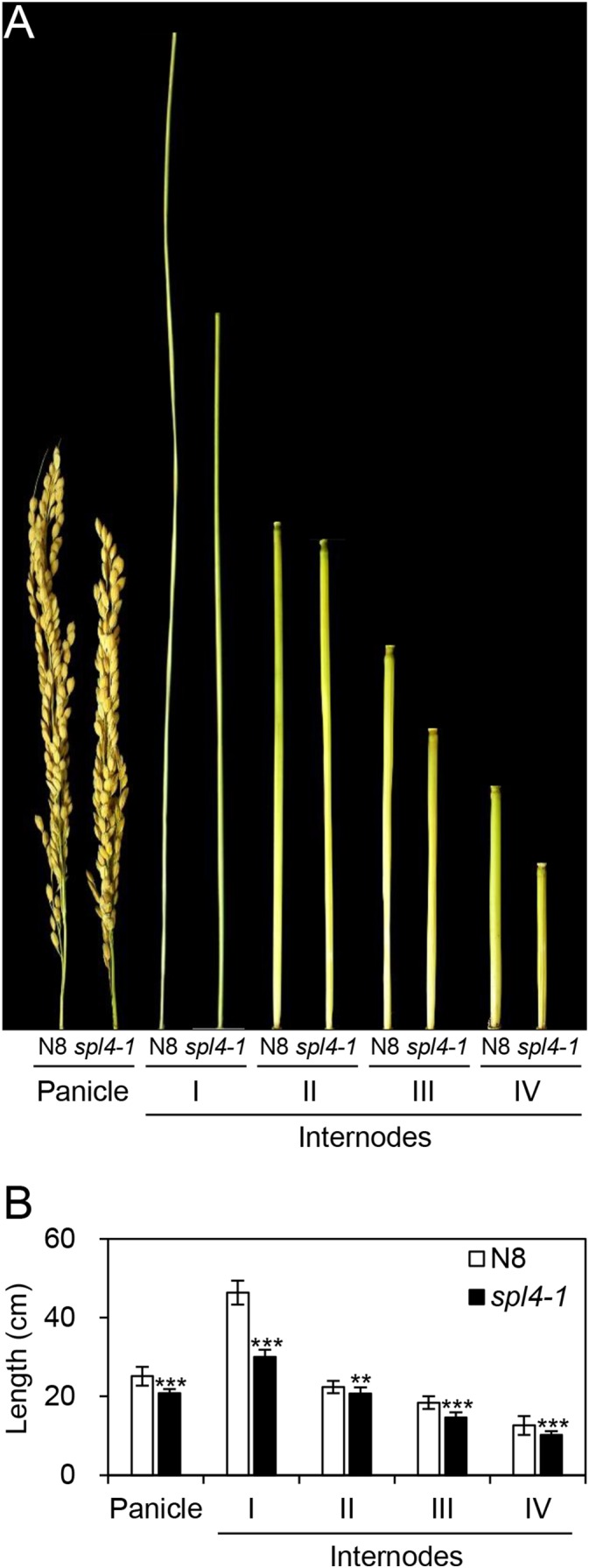
Internode length of the *spl4-1* mutant. The length of panicles and internodes was measured in wild-type Norin 8 (N8) and the *spl4-1* mutant grown in the field for 160 d after sowing. The picture **(A)** and the length **(B)** of panicles and internodes. Mean and standard deviations of all agronomic traits were obtained from twenty plants. Asterisks indicate statistically significant differences between N8 and *spl4-1* plants according to Student’s *t*-test (^∗∗^*P* < 0.01, ^∗∗∗^*P* < 0.001). The plant photos shown are representative of three independent observations.

**FIGURE 3:**
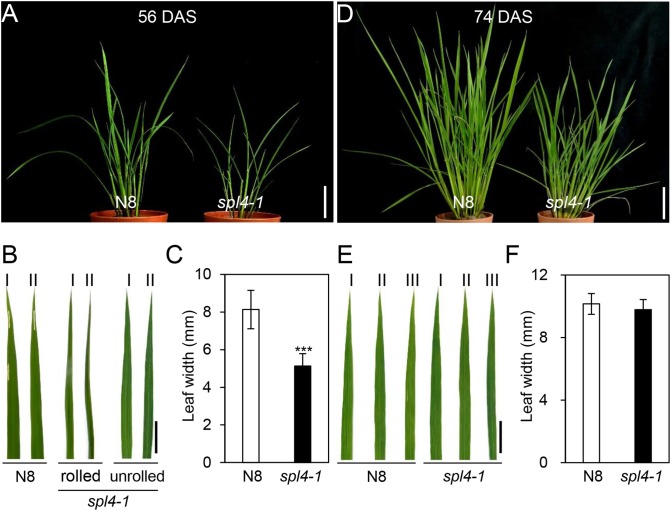
The leaves of *spl4-1* mutant temporarily rolled before lesion formation. The pictures of the N8 and *spl4-1* plants and leaves were taken of plants grown in the field at 56 days after sowing (DAS) **(A–C)** and 74 DAS **(D–F)**. **(A,D)** Plant phenotype of wild-type Norin 8 (N8, *left*) and the *spl4-1* mutant (*right*). White scale bars = 5 cm. **(B,E)** Representative leaves of N8 and *spl4-1* plants. The Roman numerals I, II, and III represent the first, second, and third leaves from the top of the plants, respectively. Black scale bars = 2 cm. **(C,F)** The leaf width was measured in N8 and *spl4-1* plants. Mean and standard deviations were obtained from more than three biological replicates. Asterisks indicate a statistically significant difference between N8 and *spl4-1* plants according to Student’s *t*-test (^∗∗∗^*P* < 0.001). The plant photos shown are representative of three independent observations.

The *spl4-1* mutant was first isolated from the γ ray-treated lines of Norin 8 (*Oryza sativa* ssp. *japonica*, hereafter referred to as N8). When N8 and *spl4-1* plants were grown in the paddy field (Suwon, South Korea, 37°N latitude), autonomous lesions began to appear in the second leaves of the *spl4-1* mutant at the maximum tillering stage (87 days after sowing, DAS) (Figures 1A,B), and then expanded throughout the entire leaf at the heading stage (118 DAS), except for the flag leaves (Figures 1C,D). In addition, the height of the *spl4-1* mutant was shorter than that of the N8 plant at the end of the grain filling stage (162 DAS) (Figures 1E,G). To investigate this height difference in more detail, we further measured the length of each internode of N8 and *spl4-1* plants. All the internodes of the *spl4-1* mutant were shorter than those of the N8 plant. The first internode of the *spl4-1* mutant was significantly shorter compared with the N8 plant (Figure 2). A number of *spl4-1* leaves rolled toward the abaxial side (Figures 3A,B), and the width of *spl4-1* leaves was narrower than that of N8 leaves at 56 DAS (Figure 3C). However, the narrow phenotype of *spl4-1* leaves disappeared and eventually became similar to the width of N8 leaves at 74 DAS (Figures 3D–F). Finally, we found that the greenness of *spl4-1* leaves persisted much longer than that of N8 leaves around the grain harvest stage (162 DAS) (Figure 1E). Consistent with this phenotype, the total chlorophyll contents of *spl4-1* leaves were higher than those of N8 leaves (Figure 1H). These results indicated that *SPL4* functions in multiple aspects of plant development including leaf senescence.

### Reactive Oxygen Species (ROS) Accumulate in the *spl4-1* Leaves

**FIGURE 4:**
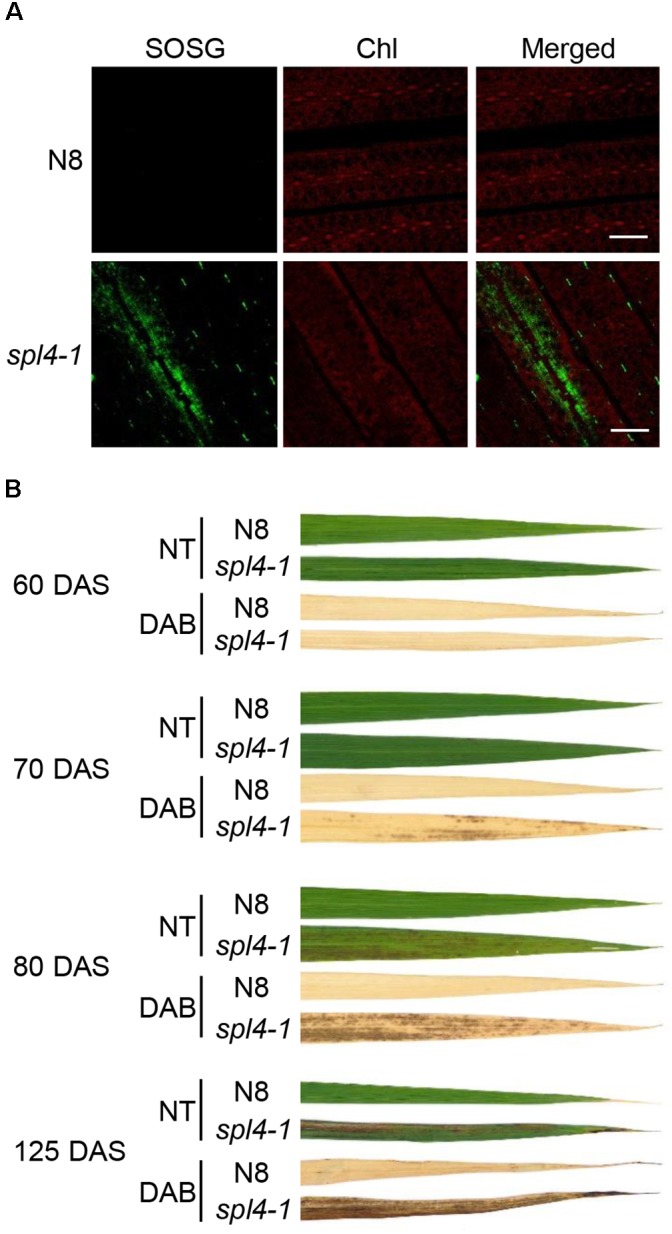
ROS accumulation in the *spl4-1* mutant. **(A)** Visualization of singlet oxygen (^1^O_2_) detected by the SOSG fluorescent probe. The leaves of wild-type Norin 8 (N8) and the *spl4-1* mutant were sampled at 133 d after sowing (DAS). The fluorescence of SOSG is green and chlorophyll (Chl) auto-fluorescence is red. Scale bars =5 μm. **(B)** DAB staining for hydrogen peroxide (H_2_O_2_) (dark brown). The leaves of N8 and *spl4-1* plants were obtained at 60, 70, 80, and 125 DAS. NT, not treated. These experiments were repeated twice with similar results.

Accumulation of ROS has been observed in the lesions of several LMMs (Chen et al., 2012; Wang et al., 2015). To investigate whether the lesions of *spl4-1* leaves is due to excessive ROS accumulation, singlet oxygen (^1^O_2_), and hydrogen peroxide (H_2_O_2_) were examined using singlet oxygen sensor green reagent (SOSG), and 3,3’-diaminobenzidine (DAB), respectively. The observation of green fluorescence indicated that ^1^O_2_ was highly accumulated in the flag leaves of 3-month-old *spl4-1* mutants, but not in those of N8 plants (Figure 4A). Little H_2_O_2_ was found in the leaf blades of the *spl4-1* mutant before the onset of lesion formation at 60 DAS. However, the red-brown precipitates accumulated when the lesions spread throughout the leaf blades of the *spl4-1* mutant at 70, 80, and 125 DAS (Figure 4B). This result suggests that, similar to other LMMs, the formation of autonomous lesions in *spl4-1* leaves is closely associated with accumulation of excess ROS.

### Map-Based Cloning of the *SPL4* Locus

**FIGURE 5:**
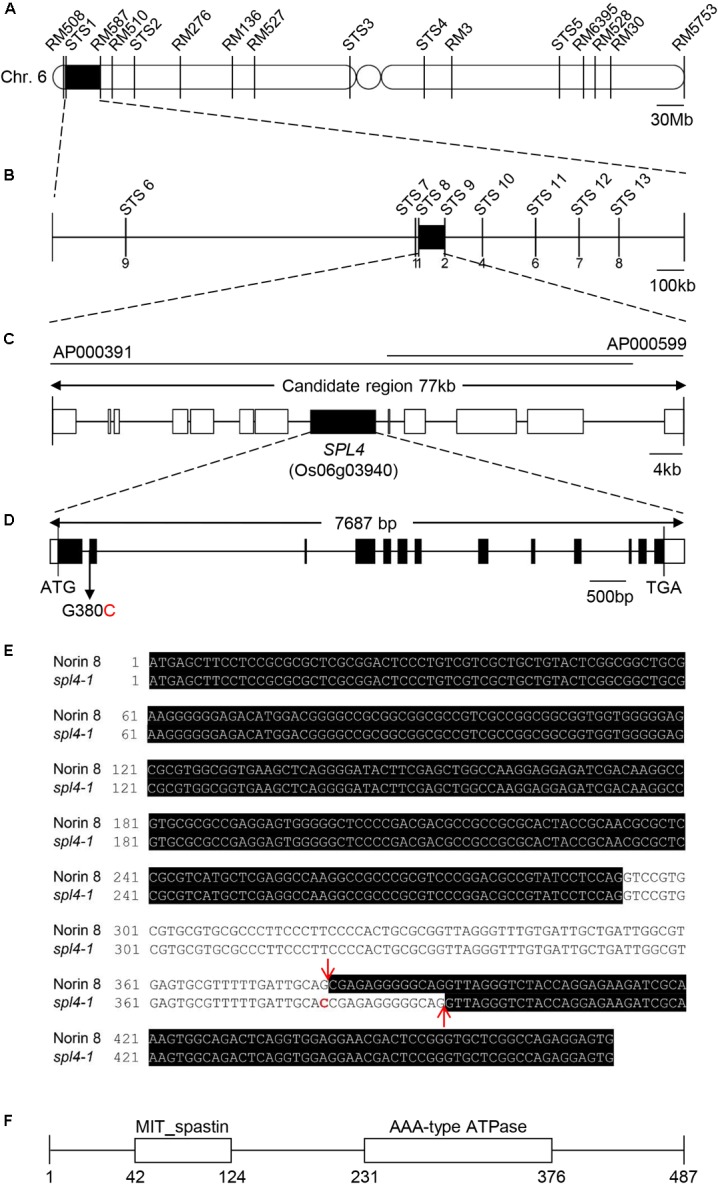
Map-based cloning of the *SPL4* locus. **(A)** Physical mapping of the *SPL4* locus. The *SPL4* locus was initially mapped to a 1.7-Mb region between two markers, STS1 and RM587, on the short arm of chromosome 6. **(B)** Fine mapping of the *SPL4* locus. The locus was further mapped within a 77-kb region between the STS8 and STS9 markers. Numbers below the line indicate the number of F_3_ recombinants at the marker regions. **(C)** Candidate genes in the 77-kb region. **(D)** The G to C substitution in *SPL4* in the *spl4-1* mutant. Black and white bars represent the exon and untranslated region, respectively. The black line represents the intron. The black arrow indicates the G to C substitution position in the *spl4-1* mutant. **(E)** *SPL4* nucleotide sequence. The black shading represents the *SPL4* mRNA. Red arrows and the red character indicate the splicing sites and the G to C substitution, respectively. **(F)** Domain structure of SPL4 containing the MIT_spastin and AAA-type ATPase domains. Numbers indicate the amino acid position of SPL4.

The *spl4-1* mutation is a single recessive allele whose locus was previously mapped to an interval of 9.6 cM on chromosome 6 (Iwata et al., 1978). Mapping of the 100 F_2_ individuals exhibiting spontaneous lesions derived from a cross between the *spl4-1* mutant and Milyang23 (a Tongil-type *indica/japonica* hybrid cultivar) initially delineated the *SPL4* locus to a 1.7-Mb region on chromosome 6 between the STS1 and RM587 markers (Figure 5A). Using 198 F_3_ individuals with the spontaneous lesion phenotype, the *SPL4* locus was fine-mapped to a 77-kb region flanked by the STS8 and STS9 markers (Figure 5B). Thirteen putative open reading frames were predicted within the candidate region according to the Rice Annotation Project Database^3^ (Figure 5C). To find single nucleotide polymorphisms (SNPs), we sequenced the genomic DNA extracted from *spl4-1* leaves using a whole-genome resequencing approach. Comparison of the sequence of the candidate region between Nipponbare (*Oryza sativa* ssp. *japonica*) and the *spl4-1* mutant revealed that a single nucleotide substitution (G to C) at the end of the 1st intron resulted in aberrant splicing of the intron of Os06g03940 (Figure 5D).

**FIGURE 6:**
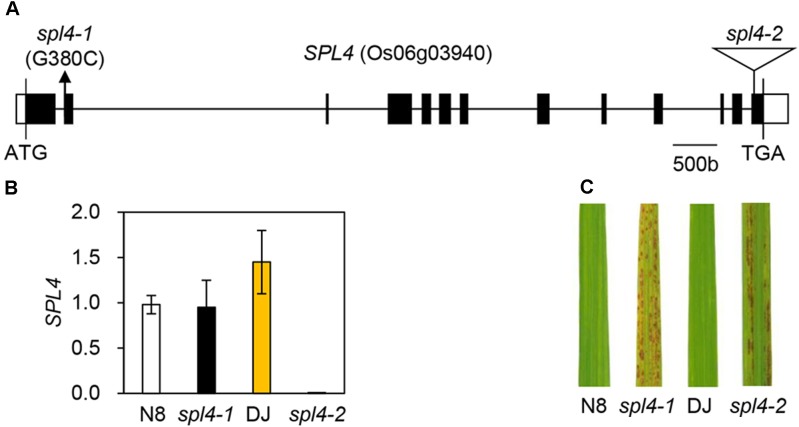
Phenotype of the T-DNA insertion *spl4-2* mutant. **(A)** Schematic diagram depicting the position of the T-DNA insertion in the *SPL4* locus (Os06g03940). Black and white bars represent the exon and untranslated region, respectively. The black line represents the introns. The black arrow and open triangle indicate the location of the *spl4-1* mutation and the *SPL4* T-DNA insertion (*spl4-2*, PFG_3A-16679), respectively. **(B)** The mutation of *SPL4* was verified in the leaves of 2-month-old wild-type plants (N8 and DJ) and *spl4* mutants. The transcripts levels of *SPL4* were determined by RT-qPCR analysis and normalized to that of *OsUBQ5*. Mean and standard deviations were obtained from more than three biological replicates. **(C)** Comparison of the formation of autonomous lesions between wild-type plants (N8 and DJ) and *spl4* mutants grown in the field at 118 days after sowing. These experiments were repeated twice with similar results.

When comparing the *SPL4* cDNA sequences between N8 and *spl4-1* plants, we found that *spl4-1* mRNA had a 13-bp deletion due to a change in the alternative splicing acceptor (Figure 5E). *SPL4* comprised 13 exons that encoded a 487-amino acid (aa) protein including a MIT_spastin domain, and subsequently an AAA-type ATPase domain (Figure 5F). To confirm that the *spl4-1* mutant phenotype is caused by loss of function of SPL4, we obtained a T-DNA insertion mutant (hereafter referred to as *spl4-2*) that contains the T-DNA insertion in exon 13 of Os06g03940 (Figure 6A). Reverse transcription quantitative PCR (RT-qPCR) analysis showed that the *spl4-2* mutant lacked *SPL4* transcript unlike its parental rice cultivar “Dongjin” (Korean *japonica* rice cultivar, hereafter referred to as DJ), while the *spl4* transcript in the *spl4-1* mutant with the 13-bp deletion was transcribed as much as the *SPL4* transcripts in the N8 plants (Figure 6B). Consistent with the phenotypic observation of the *spl4-1* mutant, the leaf blades of the *spl4-2* mutant contained spontaneous lesions (Figure 6C). Taken together, these results indicated that a null mutation of the *SPL4* locus results in the LMM phenotype.

### Loss of Function of *SPL4* Results in Delayed Leaf Yellowing Under Dark-Induced Senescence Conditions

**FIGURE 7:**
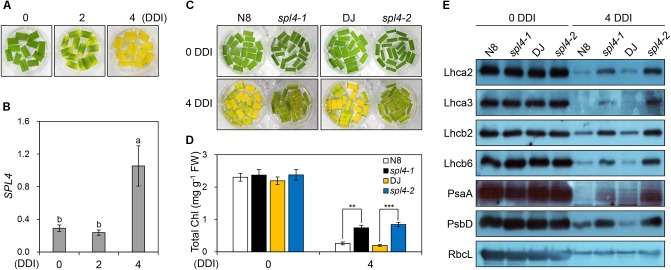
The *spl4* mutants exhibit delayed leaf yellowing during dark-induced senescence. **(A,B)** Detached leaves of 2-month-old wild-type Dongjin (DJ) grown in the field were incubated on 3 mM MES (pH 5.8) buffer at 28°C with the abaxial side up in complete darkness. The leaf yellowing phenotype **(A)** and expression profile of *SPL4* **(B)** were determined at 0, 2, and 4 days after dark incubation (DDI). The transcript levels of *SPL4* were determined by RT-qPCR analysis and normalized to that of *OsUBQ5*. Different letters indicate significant differences according to one-way ANOVA and Duncan’s least significant range test (*P <* 0.05). **(C–E)** Detached leaves of Norin 8 (N8), DJ, and the *spl4* mutants (*spl4-1* and *spl4-2*) grown in the field for 2 months were subjected to dark conditions as shown in **(A)**. The leaf yellowing phenotype **(C)** and total chlorophyll contents **(D)** were observed at 0 and 4 DDI. **(E)** An immunoblot assay was performed using antibodies against photosynthetic proteins (Lhca2, Lhca3, Lhcb2, Lhcb6, PsaA, and PsbD) and the large subunit of rubisco (RbcL). Mean and standard deviations were obtained from more than three biological replicates. Different letters indicate significant differences according to one-way ANOVA and Duncan’s least significant range test (*P* < 0.05). Asterisks indicate statistically significant differences between the wild-type plants and *spl4* mutants according to Student’s *t*-test (^∗∗^*P* < 0.01, ^∗∗∗^*P* < 0.001). These experiments were repeated twice with similar results. Chl, chlorophyll; FW, fresh weight.

The observation of more green pigments in the leaves of the *spl4-1* mutant than in the leaves of N8 plants at 162 DAS (Figure 1H) suggested that *SPL4* plays an important role in the regulation of leaf senescence. *SPL4* mRNA levels were dramatically upregulated in the fully senescing flag leaves of DJ plants at 162 DAS (Supplementary Figure S1). To analyze the leaf senescence phenotype more effectively, we used detached leaves and artificially induced senescence by a dark treatment at 28°C (Kim et al., 2006; Kong et al., 2006). When the green leaves detached from 4-week-old DJ plants turned yellow, at 4 days after dark incubation (DDI; Figure 7A), the expression levels of *SPL4* also increased rapidly (Figure 7B).

To explore the potential role of *SPL4* in leaf senescence, the leaves of 4-week-old *spl4-1* and *spl4-2* mutants and the parental cultivars N8 and DJ, respectively, were subjected to dark incubation. The green color of the leaves of both *spl4* mutants was retained much longer than that of the parental cultivars at 4 DDI (Figure 7C). Consistent with this observation, the total chlorophyll contents of both *spl4* mutants were significantly higher than those of the parental cultivars at 4 DDI (Figure 7D). To compare the levels of photosynthetic proteins between the plants at 0 and 4 DDI, we performed western blotting with antibodies against core proteins (PsaA and PsbD) of photosystem I (PSI) and PSII, and antenna proteins of the light-harvesting complexes (Lhca2, Lhca3, Lhcb2, and Lhcb6). The levels of the large subunit of Rubisco (RbcL), which is involved in carbon fixation, were also examined. While there was no remarkable difference in the levels of photosynthetic proteins between the parental cultivars and the *spl4* mutants at 0 DDI, the levels of all proteins were much higher in the *spl4* mutants than those in the parental cultivars at 4 DDI (Figure 7E). These results indicated that the mutation of *SPL4* results in delayed leaf yellowing under both natural and dark-induced senescence conditions.

### Cytoplasmic Localization of SPL4

**FIGURE 8:**
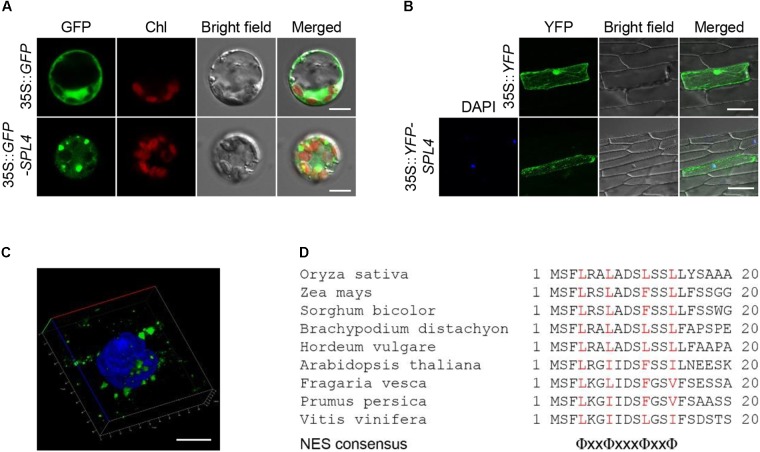
Subcellular localization of SPL4. **(A–C)** SPL4 fused with fluorescence proteins was transiently expressed in rice protoplasts **(A)** and onion epidermal cells **(B,C)** and observed by fluorescence microscopy. **(A)** The upper and lower panels show the localization of GFP and GFP-SPL4 in a rice protoplast, respectively. Chl, auto-fluorescence of chlorophyll. Scale bars = 5 μm. **(B)** The upper panels show the localization of yellow fluorescent protein (YFP) as a control and the lower panels show the localization of YFP-SPL4 in onion cells. DAPI, blue-fluorescent stain of DNA. Scale bars = 100 μm. **(C)** Enlargement of the DAPI-merged YFP-SPL4 signal shown in **(B)**. Scale bar = 10 μm. The confocal images shown are representative of three independent observations. **(D)** Sequence analysis of putative NESs derived from plant spastin proteins. Red characters indicate the conserved hydrophobic amino acids, Φ = L, I, F, V, or M. x = any amino acid.

To examine the subcellular localization of SPL4, we generated a transgenic construct in which the coding region of *SPL4* was fused in-frame with the 3′-terminus of the *green fluorescent protein* (*GFP*) reporter gene and performed a transient expression assay using rice protoplasts. The GFP signal from the *35S::GFP-SPL4* construct was observed in the cytoplasm, whereas GFP alone showed ubiquitous distribution throughout the cell (Figure 8A). To definitively identify the localization of SPL4, we generated a construct expressing a yellow fluorescent protein (YFP)-SPL4 recombinant protein and introduced this construct into onion (*Allium cepa*) epidermal cells using a bombardment-mediated transformation method. The signal of YFP-SPL4 was punctate in the cytoplasm on the periphery of the nucleus, whereas YFP alone was observed throughout the cell (Figure 8B).

To observe the speckles in more detail, we zoomed in on the DAPI-stained nucleus where the punctate structures overlapped. The enlargement indicated that the localization of YFP-SPL4 is similar to that of animal spastin spots in the cytoplasm, as previously reported (Figure 8C; Claudiani et al., 2005). Proteins that translocate to the cytoplasm from the nucleus have a conserved nuclear export signal (NES) sequence, Φ-x(2,3)-Φ-x(2,3)-Φ-x-Φ, where Φ is L, I, F, V, or M, and x is any amino acid (Bogerd et al., 1996; la Cour et al., 2003). Human spastin has three putative NES sequences that contribute to its subcellular localization in the cytoplasm (Claudiani et al., 2005). Based on the comparison of conserved plant spastin sequences in several plant species (Fekih et al., 2015), we found that a putative NES consensus, Φ-x2-Φ-x3-Φ-x2-Φ, was highly conserved in the N-terminal region of SPL4 between amino acids 4 and 14 (Figure 8D; Kosugi et al., 2008). Thus, it is possible that *SPL4* encodes a spastin protein localized in the cytoplasm.

### Defective Grain Yield of the *spl4-1* Mutant

**FIGURE 9:**
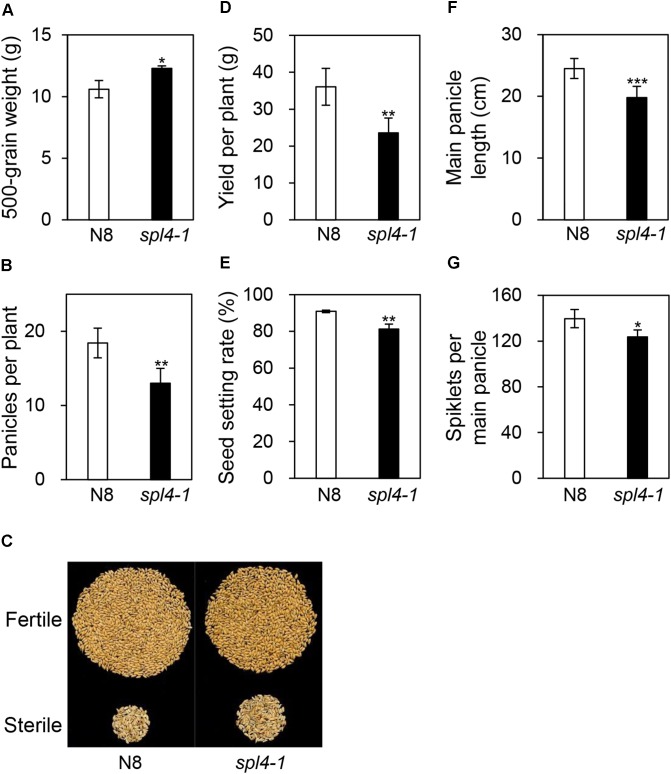
Agronomic traits of the *spl4-1* mutant. Agronomic traits were compared between wild-type Norin 8 (N8) and the *spl4-1* mutant grown in the field for 160 d after sowing. **(A)** 500-grain weight. **(B)** Number of panicles per plants. **(C)** Picture of fertile and sterile seeds from N8 and *spl4-1* plants. The seed photo shown is representative of twenty independent measurements. **(D)** Yield per plants. **(E)** Seed setting rate. **(F)** Length of main panicle. **(G)** Number of spikelets per main panicle. Mean and standard deviations of all agronomic traits were obtained from twenty plants. Asterisks indicate a statistically significant difference between N8 and *spl4-1* plants according to Student’s *t*-test (^∗^*P* < 0.05, ^∗∗^*P* < 0.01, ^∗∗∗^*P* < 0.001).

To examine whether the *spl4-1* mutation affects grain production, we evaluated the yield components, including 500-grain weight, grain yield per plant, panicle length, panicles per plant, spikelets per main panicle, and the seed setting rate, in N8 and *spl4-1* plants grown in the field. Interestingly, among the yield components, the *spl4-1* mutants showed a 12% increase in the 500-grain weight compared with N8 plants (Figure 9A). However, the *spl4-1* mutants had a relatively low seed setting rate and number of panicles per plant compared with N8 plants, resulting in lower grain yield per plant (Figures 9B–E). Moreover, the shorter length of the main panicle in the *spl4-1* mutant caused a decrease of the number of spikelets per main panicle compared to N8 plants (Figures 9F,G). Thus, the increase of 500-grain weight did not lead to an improvement of grain yield in *spl4-1* mutants.

## Discussion

The mutation of the *SPL4* locus has been previously reported as *lesion mimic resembling* (*lmr*) and *lesion resembling disease 6-6* (*lrd6-6*) in rice (Fekih et al., 2015; Zhu et al., 2016). These mutants, lacking the AAA-type ATPase activity, exhibited an enhanced resistant phenotype against the rice blast fungus (*Magnaporthe oryzae*) and bacterial blight (*Xanthomonas oryzae* pv. *oryzae*) by inducing pathogenesis-related genes and promoting the biosynthesis of antimicrobial compounds. The studies of most LMMs have been focused on defense mechanisms against pathogen attacks due to the phenotypic similarity between lesion mimicry and the HR (Qiao et al., 2010; Feng et al., 2013; Li et al., 2014). In this study, we characterized the s*pl4-1* mutant and report a novel function of SPL4 in the regulation of leaf senescence. Consistent with most LMMs isolated from various plant species (Hoisington et al., 1982; Wolter et al., 1993; Dietrich et al., 1994; Badel et al., 2006; Kim et al., 2009), the *spl4-1* mutants exhibit autonomous lesions along with excessive ROS accumulation (Figures 1, 4).

Based on map-based cloning and a domain search in the NCBI database^4^, the conserved amino acid sequences of SPL4 were predicted to include the MIT_spastin, and AAA-type ATPase domains (Figure 5F). Notably, the rice genome does not contain any other gene that is homologous to the MIT_spastin domain of SPL4. Spastin is well-studied in animal cells because of its important role in HSP disease (Crosby and Proukakis, 2002). Human spastin, encoded by *SPG4*, localizes in cytoplasmic spots on the periphery of the nucleus (Claudiani et al., 2005). The localization of SPL4 in plant cells was similar to that of SPG4 in animals. Moreover, when YFP-SPL4 was transiently expressed in onion epidermal cells, fluorescent signals were detected in the cytoplasm around the nucleus (Figures 8B,C). These observations indicate that *SPL4* encodes a putative plant spastin, possibly functioning in inhibition of lesion formation during leaf development.

The NES is the essential conserved amino acid residues that allow the protein to export from the nucleus to the cytoplasm. There are three putative NES sequences in human spastin (Claudiani et al., 2005). Chromosome region maintenance 1 (CRM1), a member of the importin β superfamily, recruits the CRM1-NES cargo-RanGTP complex, and subsequently recognizes the NES consensus sequence of human spastin to facilitate the transportation (Ossareh-Nazari et al., 2001; Fornerod and Ohno, 2002; Claudiani et al., 2005; Hutten and Kehlenbach, 2007). In Arabidopsis, EXPORTIN 1 (AtXPO1/AtCRM1) forms the export complex with a plant cofactor, RAN1. This complex interacts with NES residues that are derived from animal proteins and transports the target proteins from the nucleus to the cytoplasm (Haasen et al., 1999). NES consensus sequences are typically composed of hydrophobic conserved residues separated by a variable number of amino acids, given by Φ-x(2,3)-Φ-x(2,3)-Φ-x-Φ. However, Kosugi et al. (2008) defined a new NES consensus sequence through screening of a *∆tys1* knockout yeast strain that can be rescued by tyrosyl-tRNA synthetase 1 (TYS1), which can anchor to random NES sequences (Kosugi et al., 2008). Among the six NES consensus patterns identified from Kosugu’s selection system, two NES sequences are inconsistent with the traditional rule; one is Φ-x-Φ-x2-Φ-x-Φ and the other is Φ-x2-Φ-x3-Φ-x2-Φ (Kosugi et al., 2008). These diverse NESs may reflect the wide spectrum of binding specificity of CRM1. Interestingly, both monocots and dicots have highly conserved spastin proteins (Fekih et al., 2015). Through our attempt to find the NES sequences in plant spastin proteins, we defined the plant-specific NES sequence as Φ-x2-Φ-x3-Φ-x2-Φ (Figure 8D), which differs from that of human spastin. Thus, our results suggest a putative plant-specific NES sequence that may contribute to the cytoplasmic localization of SPL4.

Leaf yellowing is a visual marker for estimating the degree of leaf senescence (Lim et al., 2007). The *spl4-1* mutant exhibited delayed leaf yellowing compared to its parental *japonica* rice cultivar (Norin 8) under both natural and dark-induced senescence conditions (Figures 1F,H, 7C,D). Based on these observations, we hypothesized that alteration of MT arrays, which is regulated by MT-associated proteins (MAPs) and MT-severing proteins including spastin (Hazan et al., 1999), katanin (McNally and Vale, 1993) and fidgetin (Cox et al., 2000), is a key factor for the regulation of leaf senescence. Keech et al., 2010 showed that the MT network in epidermal and mesophyll cells of Arabidopsis leaves are degraded during natural and dark-induced leaf senescence. This MT destabilization is closely connected with the expression of genes that encode the MAP proteins. While the genes encoding the MT-stabilizing proteins (MAP65 family and MAP70-1) were repressed during natural and dark-induced leaf senescence, transcripts of *MAP18*, encoding a MT-destabilizing protein, are strongly upregulated in senescing Arabidopsis leaves (Keech et al., 2010). Thus, MT plasticity is closely linked to the stability of epidermal and mesophyll cells that are involved in photosynthesis.

Although expression of *SPL4* significantly increased in wild-type leaves in the dark-induced senescence treatment (Supplementary Figure S1 and Figures 7A,B), the underlying regulatory mechanisms responsible for leaf senescence remain unknown. The expression of katanin p60 subunit genes, encoding proteins of another MT-severing family, is significantly upregulated under dark-induced senescence (Keech et al., 2010). Arabidopsis plants overexpressing the katanin p60 subunit (*AtKSS*) form numerous bundles of MTs, resulting from the severing of MTs by AtKSS, and then the MTs are ultimately depolymerized (Stoppin-Mellet et al., 2006). The *bot1-1* mutant, with a mutation in *BOT1* encoding katanin, survives much longer than wild-type plants (Bichet et al., 2001). Here, our analysis showed that photosynthetic proteins remained much more abundant in the *spl4* mutants than in the parental cultivars at 4 DDI (Figure 7E). By analogy to the effects of the MT-severing protein katanin, we hypothesize that senescence-induced spastin severs the MTs of rice epidermal and mesophyll cells, followed by promoting the degradation of photosynthetic proteins.

Although ROS accumulation generally accelerates leaf senescence (Jajic et al., 2015; Lin et al., 2016), our results showed that excessive accumulation of ROS in *spl4-1* mutant does not link to the promotion of leaf yellowing during dark incubation (Figures 4, 7). Recently, Wang et al. (2015) reports that the LMM phenotype by mutation of *SPOTTED LEAF3* (*SPL3*) is due to excessive ROS. However, ABA hyposensitivity of *spl3* mutant leads to delaying leaf yellowing during both natural and dark-induced senescence. Since leaf senescence is a complex process involving numerous regulators (Guo et al., 2004; Buchanan-Wollaston et al., 2005), plants may have alternative senescence pathways that are mediated by spastin independent of ROS signaling.

Finally, mutation of *SPL4* affects plant morphology, including short internodes (Figure 2). In maize, internodal cells originate from a portion of undifferentiated cells which have randomly arranged cortical MTs (Nemoto et al., 2004). These random cortical MTs are transversely reoriented in incipient internode, followed by rearranging to longitudinal direction during internode elongation. The severing activity of spastin may contribute to reorganization of cortical MTs (Shibaoka, 1994). In this scenario, it is highly possible that impairment of spastin function in *spl4-1* mutant inhibit the polarization of cortical MTs in internode, resulting in semi-dwarfism.

## Author Contributions

GS and C-TK performed all the experiments. H-JK, KK, and N-CP designed the research. S-HK assisted in the phenotypic characterization. YS and CL performed the leaf senescence analyses. GA developed the *spl4-2* mutant and provided advice about the manuscript. GS, C-TK, KK, and N-CP wrote and edited the manuscript. All authors have read and approved the content of the final manuscript.

## Conflict of Interest Statement

The authors declare that the research was conducted in the absence of any commercial or financial relationships that could be construed as a potential conflict of interest.

## Footnotes

1 http://signal.salk.edu/cgi-bin/RiceGE

2 http://www.gramene.org

3 http://rice.plantbiology.msu.edu

4 https://www.ncbi.nlm.nih.gov/Structure/cdd/wrpsb.cgi
