# Supplementary material for: The Rice SPOTTED LEAF4 (SPL4) Encodes a Plant Spastin That Inhibits ROS Accumulation in Leaf Development and Functions in Leaf Senescence
Source: Front Plant Sci. 2019 Jan 7;9:1925. doi: 10.3389/fpls.2018.01925 (PMC6330318; doi:10.3389/fpls.2018.01925)
Supplement: Supplementary file 1 [file Data_Sheet_1.PDF]

Supplemental Information

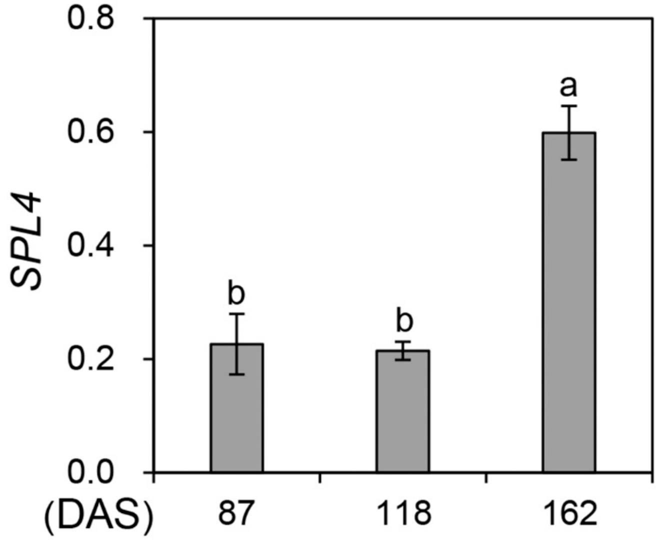

FIGURE S1. Expression profile of *SPL4* in the field conditions.

Total RNA was isolated from the attached leaves of wild-type “Dongjin” at 87, 118, and 162 days after sowing (DAS). The transcript levels of *SPL4* were determined by real-time RT-qPCR analysis and normalized to that of *OsUBQ5*. Means and standard deviations were obtained from more than three biological replicates. Different letters indicate significant differences according to one-way ANOVA and Duncan’s least significant range test ($P < 0.05$). These experiments were repeated twice with similar results.

TABLE S1. Primers used in this study.

	Forward primer (5'→3')	Reverse primer(5'→3')
A. Primers for map-based cloning		
RM508	GGATAGATCATGTGTGGGGG	ACCCGTGAACCACAAAGAAC
STS1	AGCTCAATATCAGGCAAGCAG	AAATGACACAGTTGACCTTTTGAA
RM587	ACGCGAACAAATTAACAGCC	CTTTGCTACCAGTAGATCCA
RM510	AACCGGATTAGTTTCTCGCC	TGAGGACGACGAGCAGATTC
STS2	ATAGCAGGGTTGCCTTCTCA	GGGGCCATAACCCTAGACAT
RM276	CTCAACGTTGACACCTCGTG	TCCTCCATCGAGCAGTATCA
RM136	GAGAGCTCAGCTGCTGCCTC	GAGGAGCGCCACGGTGTACG
RM527	GGCTCGATCTAGAAAATCCG	TTGCACAGGTTGCGATAGAG
STS3	CCCCTTCATCATTGCAACTT	AGTCTCTCCATCACCCGTCT
STS4	CGTCGTACCCCTCATGTCTT	CACGCAATCTGTGTAATTAGTTTTT
RM3	ACACTGTAGCGGCCACTG	CCTCCACTGCTCCACATCTT
STS5	TAATGGTTGCAATGGGGCCT	GGCATAGTGCTCCTCTAGGC
RM6395	CTTCGGCTTCTGAACTAGCG	CAGTGCCGATGATCCTCTTC
RM528	GGCATCCAATTTTACCCCTC	AAATGGAGCATGGAGGTCAC
RM30	GGTTAGGCATCGTCACGG	TCACCTCACCACACGACACG
RM5753	AACATGCTCAACTTCTGGGC	GCTAGGTACGATCCAGCTGC
STS6	ATCAGAGTGAGGTAATCTGGACC	ACCATTTGATTTAGGTATATGAA
STS7	TAATGTCTTGGGTTGGGAATGGC	ATGCACTGCTCTGGCACACAAGT
STS8	GATTCATACCTTCACTTTGCGAT	AGCAAACTATGCAACTAACTGCA
STS9	TCAGTGTCCTTTGAACCATCTCAT	CTGCAGAAATGAAGTGACAGTTA
STS10	TGGATTAGTTCATTG32212.0TGCTATGA	AGAAGATGTCTTCGTCAGAGTTCT
STS11	AAGTATTAGCGTTAGTCGCCGG	TTGAAACAGTTTGACTTTGACCA
STS12	AAACTGGAGCAGATAACATGGCA	AGGATCGCATTGCACCGCTAGCT
STS13	ACCTTTCAGGAGCTATACCATTTGG	AAGTTTAGTTGATGCTTCCTTTTGT

B. Primers for vector construction		
spl4	ATGAGCTTCCTCCGCGCGCTCGCG	ACTTGAACCAAATTCTTCGTTCC
C. Primers for verification of *spl4-2* T-DNA insertion		
PFG 3A-16679	GCATTTTTAAAGCGTGGAGC	GATCTTGAAAGGCTTGCTGC
D. Primers for RT-qPCR analysis		
SPL4	TGATCTTGAAAGGCTTGCTGCAG	GCTTGGCCTAATCACAGTCATGG
OsUBQ5	ACCACTTCGACCGCCACTACT	ACGCCTAAGCCTGCTGGTT
